# Training Set Selection for the Prediction of Essential Genes

**DOI:** 10.1371/journal.pone.0086805

**Published:** 2014-01-22

**Authors:** Jian Cheng, Zhao Xu, Wenwu Wu, Li Zhao, Xiangchen Li, Yanlin Liu, Shiheng Tao

**Affiliations:** 1 College of Life Sciences and State Key Laboratory of Crop Stress Biology in Arid Areas, Northwest A&F University, Yangling, Shaanxi, China; 2 Bioinformatics Center, Northwest A&F University, Yangling, Shaanxi, China; 3 Key Laboratory of Food Safety Research, Institute for Nutritional Sciences, Shanghai Institutes for Biological Sciences, Chinese Academy of Sciences, University of Chinese Academy of Sciences, Shanghai, China; 4 College of Science, Northwest A&F University, Yangling Shaanxi, China; 5 College of Wine, Northwest A&F University, Yangling Shaanxi, China; Technische Universität Dresden, Medical Faculty, Germany

## Abstract

Various computational models have been developed to transfer annotations of gene essentiality between organisms. However, despite the increasing number of microorganisms with well-characterized sets of essential genes, selection of appropriate training sets for predicting the essential genes of poorly-studied or newly sequenced organisms remains challenging. In this study, a machine learning approach was applied reciprocally to predict the essential genes in 21 microorganisms. Results showed that training set selection greatly influenced predictive accuracy. We determined four criteria for training set selection: (1) essential genes in the selected training set should be reliable; (2) the growth conditions in which essential genes are defined should be consistent in training and prediction sets; (3) species used as training set should be closely related to the target organism; and (4) organisms used as training and prediction sets should exhibit similar phenotypes or lifestyles. We then analyzed the performance of an incomplete training set and an integrated training set with multiple organisms. We found that the size of the training set should be at least 10% of the total genes to yield accurate predictions. Additionally, the integrated training sets exhibited remarkable increase in stability and accuracy compared with single sets. Finally, we compared the performance of the integrated training sets with the four criteria and with random selection. The results revealed that a rational selection of training sets based on our criteria yields better performance than random selection. Thus, our results provide empirical guidance on training set selection for the identification of essential genes on a genome-wide scale.

## Introduction

As a minimal gene subset in organisms, essential genes are required for survival, development and fertility [1], [2]. Identifying such genes can aid in understanding the primary structures of complex gene regulatory networks in a cell [3]–[5], elucidating the relationship between genotype and phenotype [6], [7] and discovering potential drug targets in novel pathogens [8]–[10]. In addition, they can be useful in re-engineering microorganisms [11], [12], particularly for investigating the causes of human diseases [13], [14].

Prediction and identification of essential genes are done primarily by experimental and computational techniques. Experimental techniques randomly or systematically inactivate potential essential genes and assess their essentiality based on the effects on organisms [15]–[18]. However, in some organisms such as mammals, experimental techniques are time-consuming and expensive. In addition, the degree of gene essentiality varies under different growth conditions [6], [19]. For this reason, computational techniques are used, often in combination with experimental techniques, to both predict and identify essential genes. Known essential genes from various microorganisms provide instructional and training materials for computational studies. In addition, genome sequences obtained by high-throughput sequencing provide relevant information for investigating the minimal subset of genes in various organisms. Notably, recent development in bioinformatics has significantly advanced the computational tools and resources available to investigate essential genes.

Several prediction models have been developed to identify essential genes *in silico*. One of the simplest models utilizes known essentiality of homologous genes to predict new essential genes [20]–[23]. Although this model is generally reliable, two limitations have been observed. First, conserved orthologs between species account for a small portion of a genome [24]. Second, orthologs in distantly related species often exhibit differences in gene regulation and function [25], leading to a potential diversity of gene essentiality. To circumvent these limitations, researchers have developed feature-based models that can be used to distinguish essential genes from non-essential ones based on the presence of features similar to those of essential genes [26]–[30].

To improve the accuracy of feature-based machine learning methods, researchers have sought to determine special gene features (e.g., network topology feature, flux deviations, and domain enrichment) that significantly correlate with gene importance [26]–[33]. In addition to feature selection, training set selection is used as an alternative strategy to improve the accuracy of machine learning methods; however, training set selection is often ignored in the absence of sufficient data. Existing knowledge of a large number of essential genes in various microorganisms provides a great opportunity to investigate training set selection and thereby improve prediction of essential genes.

**Figure 1:**
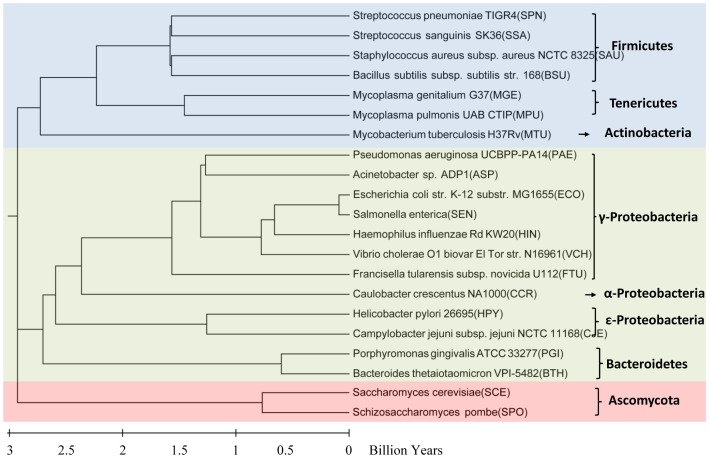
Phylogenetic tree of the 21 species. The divergence times of the 20 species were obtained from a public database TIMETREE [57] (Table S3).

In the present study, a naïve Bayes classifier was applied initially to reciprocally predict essential genes among 21 species (Figure 1, Table S1). We found that the predictive accuracy based on different training sets varied significantly. We then demonstrated that this variation could be attributed to quality of training set, growth conditions, evolutionary distance, and lifestyle. Subsequently, we investigated the performance of incomplete and integrated training sets. Our results showed that at least 10% of the total genes were necessary to achieve optimum performance. We also demonstrated that integrated training sets were more stable and accurate compared to single sets. Finally, we validated the better performance of our selective four rules for training set selection by comparing with random selection.

## Materials and Methods

### Essential gene and gene sequences

The essential genes of 21 species (Table S1) were obtained from relevant studies, as well as the Online Gene Essentiality Database (OGEE) [34] and Database of Essential Genes (DEG) [35]. The cDNA and protein sequences of the 21 species were downloaded from the NCBI server (ftp://ftp.ncbi.nih.gov/genomes/). The homologous map and proteome sequences of 417 core species were downloaded from eggNOG 3.0 [36].

### Features collection

We collected 15 features (see feature descriptions in Table 1) that are widely used in previous models. All 15 features are divided into six categories as following.

**Table 1:** Abbreviations and descriptions of selected features.

Abbreviation	Description
mE	mRNA Expression level
mEF	mRNA Express Fluctuation
Age	gene origin age
DoT	gene domain type
DoC	gene domain conservation
DC	network topology feature, degree centrality
CCo	network topology feature, clustering coefficient
CC	network topology feature, closeness centrality
BC	network topology feature, betweenness centrality
PL	protein length
CAI	codon adaptation index
NP	number of paralogs for a target gene
NS	number of species which have at least a homology for a target gene
NEH	number of essential homolog genes in other species for a target gene
NNH	number of non-essential homolog genes in other species for a target gene

- Domain properties. Essential genes are associated with basic categories of biological functions or processes [37]. Therefore, essential genes may contain some shared domains. To identify the domain of each gene in the 21 species we investigated, we first downloaded the hidden Markov models (Pfam-A.hmm) of protein domains from the Pfam database [38], and then used the Hmmer software [39] to identify the protein domain of each gene. The corresponding domain type for each gene was defined as the feature DoT. The amino acid sites within protein domains are often more important and conserved than those in other parts. Therefore, we assumed that the conservation of protein domain is a reflection of gene essentiality, and the DoC of each gene was calculated according to the ratio of the conserved domain score and the domain length.
- Protein–protein interaction (PPI) network. Network topology features have been used widely in previous studies. They indicated that essential genes tend to play topologically more important roles in protein interaction networks than non-essential genes. In our study, PPI data for the genes in 21 species were downloaded from the STRING Database [40]. Then, we used the NetworkX software package [41] to compute the four network topology features, i.e. DC, CCo, CC, and BC (see feature descriptions in Table 1).
- Genomic sequence properties. Although protein length (PL) tends to become longer through evolution [42], different natural constraints might exist on the PL between essential genes and nonessential genes. The codon usage of essential genes suffers from more evolutionary constraints than non-essential genes. We used the CodonW [43] software package to calculate codon usage, i.e. CAI.
- Homology properties. Duplicated genes are believed to often overlap in function and expression [44], and duplicates are always less likely to be essential than singletons [45]–[47]. An all-against-all BLAST search was conducted for the whole set of proteins in each of the 21 species to identify the paralogs with an E-value threshold of 10^−20^, and the number of paralogs for a target gene within each species was used as the feature NP. Four-hundred seventeen core organisms in the eggNOG database included all of the 21 species in our study. Therefore, we counted the number of species among the 417 core species that had at least one homologous gene for each target gene in 21 species (feature NS). The orthologous gene of an essential gene is highly likely to be essential as well [48]. Therefore, we calculated the numbers of essential and non-essential homologous genes, including those that are found in other species, for each target gene (NEH and NNH).
- Phyletic gene age. Chen [46] showed that older genes (i.e. genes with earlier phyletic origin) are more likely to be essential than young ones. Age was calculated according to previously described methods [46], [49] and the target genomes of *SCE* and *SPO* were divided into five taxonomic groups, i.e. species typical, Ascomycota, Opisthokonta, Eukaryota, and cellular organisms.
- Gene expression. mRNA expression data were obtained from Series GSE15352 [50] and GSE30025 [51] of the Gene Expression Omnibus (GEO) Database. The expression levels of essential genes are often higher and more stable than those of non-essential genes [52]. The average and variable coefficients of mRNA expression levels in all conditions were collected as predictors (i.e. mE and mEF).

## Results and Discussion

### Influences of different training sets on the predictive accuracy

To investigate the manner and extent by which training set selection affects predictive accuracy, we trained and reciprocally predicted essential genes in 21 organisms by using naïve Bayes classifiers. The result are presented as a 21×21 AUC matrix *M* = (*m_ij_*), where *m_ij_* is the AUC score with *i*th species as a training set and *j*th species as a testing set.

**Figure 2:**
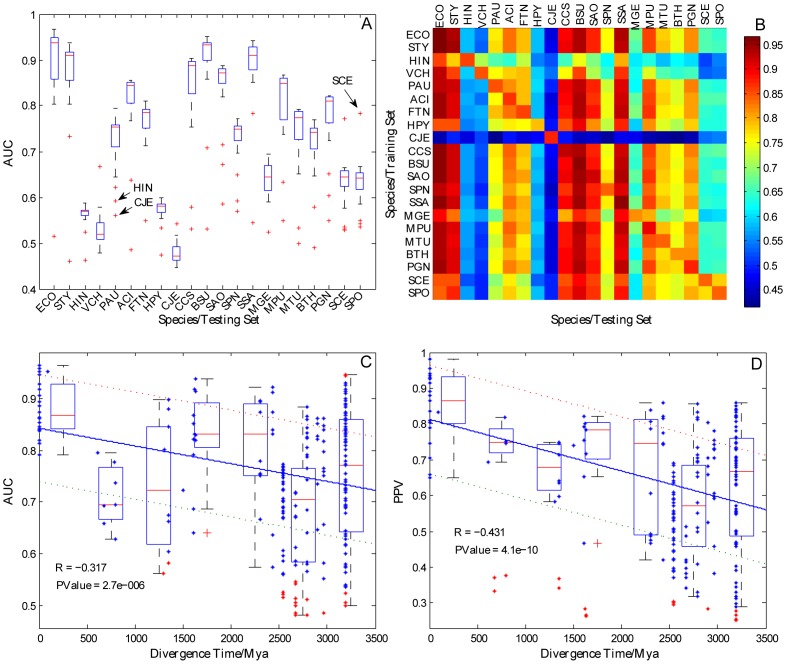
Distribution of AUC scores among 21 species and the correlation between predictive accuracy and divergence time. **A**) Variation in prediction accuracy from different training sets to the same testing set. Each boxplot displays the variation in AUC scores calculated from different training sets (in addition to the species on the X axis, the rest of the 20 species were used as the training set) relative to the same testing set (the species on the X axis). ‘+’ represents the outliers corresponding to the training sets that have significantly lower or higher predictive accuracy. **B**) Heatmap matrix of the influence of training sets on different species. Colors in each cell indicate AUC scores obtained when those species were used as the training set to predict the essential genes of the target 21 species including the speciesself. *CJE* shows the worst prediction performance when *CJE* was used as the training set. **C**) Correlation between AUC scores and divergence times. Stars refer to the AUC scores obtained from the organisms with the divergence time on the X axis, in which one species is used as a training set and the other was used as a testing set. Red stars refer to outliers that were discarded from the regression analysis. The regression line is indicated by a solid line, and error bars are indicated by dashes. The boxplot shows the variation in prediction per 500 millions of years. **D**) Correlation between PPV scores and divergence times.

Since the ratios of essential genes among organisms were different, and comparing the differences in AUC scores calculated based on the predictions from the same training set to different testing sets would not be meaningful. Therefore, we compared the AUC scores obtained from the same testing set and different training sets for each of the 21 species. Based on the AUC matrix, the variation of AUC scores from different training sets was displayed as boxplots (Figure 2A), and these variations were applied to determine the influence of different training sets on predictive accuracy. We found that the interquartile ranges (IQRs) of many species within testing sets were >0.03 (Table S2). *ECO* (*E. coli*) showed the largest IQR (0.091), demonstrating that the difference in predictive accuracy may exceed 9% when different species are selected as the training set.

Besides, some outliers were observed in the boxplots (Figure 2A), indicating that some training sets exhibited significantly lower or higher predictive accuracies than other sets. For example, the accuracy of *SCE* (*S. cerevisiae*) as the training set used to predict the essential genes of *SPO* (*S. pombe*) was significantly better than that of the other species. By contrast, use of *HIN* (*H. influenza*, AUC = 0.592), *CJE* (*C. jejuni*, AUC = 0.563), and *MGE* (*M. genitalium*, AUC = 0.621) as training sets exhibited significantly lower prediction accuracies than other species when *PAU* (*P. aeruginosa*) was used as the testing set. In summary, different training sets influenced the predictive accuracy significantly, and we explain these observed variations in the context of four factors discussed in detail below.

### Quality of training set

To elucidate why some training sets exhibited significantly lower predictive accuracy than other species, we closely examined the heatmap of AUC scores (Figure 2B). We found that *CJE*, when used as the training set, exhibited the least performance among the predictive groups (each column as a group with the same testing set in Figure 2B). Although *CJE* (which belongs to *ε*-Proteobacteria) is distantly related to other species (Figure 1), we concluded that the extremely low AUC scores were a result of systematic biases in transposon mutagenesis (e.g., insertion-site preference, unsaturated insertion mutants, or polar effects) when essential genes in *CJE* were identified [53], [54]. A similar pattern was observed when *HIN* was used as the training set to predict essential genes in *HPY*, *SCE* and *SPO*. This is attributed mainly to the fact that the essential genes of *HIN* were obtained by integrating multiple sets of experimental data by bioinformatics methods. The poor accuracy of *CJE* and *HIN* demonstrated that the transferability of essentiality annotations between species was significantly affected by the quality of the training set. In general, the quality of essential genes identified by a genome-wide set of gene deletions was better than that identified by transposon mutagenesis, RNAi, and other methods. In subsequent experiments, *CJE* was removed from our study materials and the remaining 20 species were used as the training set or the testing set.

### Difference in growth conditions

Papp [3] indicated that 18% to 34% of dispensable genes in *SCE* are not important under nutrient-rich conditions but are considered important under other conditions. Therefore, growth conditions may greatly affect predictive accuracy because of inconsistent essential gene sets in different media. For example, the growth conditions for the identification of essential genes in *ECO* and *ACI* (*Acinetobacter sp. ADP1*) were standard laboratory-rich (Luria-Bertani) media and minimal medium supplemented with succinate, respectively. Thus, many genes in *ACI* involved in the biosynthesis of important compounds are essential because these compounds are absent in minimal media. This difference in media accounts for a substantially higher percentage of essential genes in *ACI* (16%) than in *ECO* (7%). In our experiment, although *ECO* is more closely related to *ACI* than *SSA* (*S. sanguinis*), the prediction of *ECO*-*ACI* exhibited lower true positive rate (TPR = 0.60) than that of *SSA*-*ACI* (TPR = 0.63). This may be because the essential genes of *SSA* were identified in minimal medium, which is the same as that of *ACI*. Deng [30] removed 82 genes associated with biosynthesis from the *ACI* essential gene set. The refined data exhibited considerably better precision in predicting *ECO* essential genes. These results suggested that the growth conditions under which essential genes are defined in the training set should be determined because those conditions considerably affect the predictive accuracy of minimal gene subset.

### Evolutionary distance between species

Deng [30] indicated that gene essentiality can be reliably predicted in a distantly related organism. However, the predictive accuracy of essential genes in closely related organisms is higher in general than that in distantly related organisms. To investigate the manner and the extent by which evolutionary distance affects predictive accuracy, we analyzed the correlation between AUC scores and divergence times (Figure 2C). The results revealed significant negative correlation between AUC scores and divergence times (R = −0.317, p = 2.7e–6). Moreover, we found that a greater evolutionary distance between organisms resulted in a larger variation in predictive accuracy.

To obtain a more general understanding of this negative correlation [55], we selected another parameter called positive predictive value (PPV), which represents the number of genes predicted and verified as essential, to assess predictive accuracy. The PPV scores were calculated by determining the proportion of true essential genes in the first 200 genes exhibiting the highest essentiality scores. By a similar method to that used for AUC matrix, the PPV matrix was then obtained to analyze the correlation between PPV scores and divergence times (Figure 2D). PPV scores showed a stronger negative correlation with divergence times than AUC scores (R = −0.431, p = 4.1e–10). The same patterns were observed in PPV scores calculated for the first 100, 300, and 400 genes (Table S4). Furthermore, we calculated the correlation between predictive accuracy and divergence times for each species used as the testing set (Table 2). Although all of the 20 species tested showed negative correlations, only those of *HIN* (−0.563), *VCH* (−0.685), *HPY* (−0.703), *SPN* (−0.495), *MGE* (−0.707), *SCE* (−0.818), and *SPO* (−0.850) were statistically significant. This result is likely attributable to the non-uniform divergence times between species.

**Table 2:** Correlation between predictive accuracy and divergence times for each species as the testing set.

Species	*R* ^1^	P-value^2^	*R* ^3^	P-value^4^
ECO	−0.123	0.5944	−0.153	0.5207
STY	−0.084	0.7189	−0.148	0.5342
**HIN**	**−0.563**	**0.0079**	**−0.673**	**0.0011**
**VCH**	**−0.685**	**0.0006**	−0.195	0.4099
PAU	−0.358	0.1113	−0.204	0.3886
ACI	−0.072	0.7563	−0.208	0.3781
FTN	−0.264	0.2479	−0.273	0.2437
**HPY**	**−0.703**	**0.0004**	**−0.911**	**2.4E-8**
CCS	−0.098	0.6723	−0.248	0.2915
BSU	−0.248	0.2782	0.027	0.9095
SAO	−0.350	0.1196	0.001	0.9971
**SPN**	**−0.495**	**0.0225**	**−0.613**	**0.0041**
SSA	−0.267	0.2421	−0.012	0.9583
**MGE**	**−0.707**	**0.0003**	−0.405	0.0769
MPU	−0.211	0.3595	0.064	0.7878
MTU	−0.294	0.1955	−0.225	0.3397
BTH	−0.309	0.1726	−0.349	0.1310
PGN	−0.255	0.2652	−0.290	0.2147
**SCE**	**−0.818**	**6.0E-6**	**−0.862**	**1.0E-6**
**SPO**	**−0.850**	**1.1E-6**	**−0.597**	**0.0055**

Note: The first column indicates the species used as the testing set. *R* ^1^ refers to Pearson correlation coefficient between AUC scores and divergence times. P-value^2^ corresponds to the significance level of *R* ^1^. *R* ^3^ refers to Pearson correlation coefficient between PPV scores and divergence times. P-value^4^ corresponds to the significance level of *R* ^3^.

### Difference in phenotype and lifestyle

**Figure 3:**
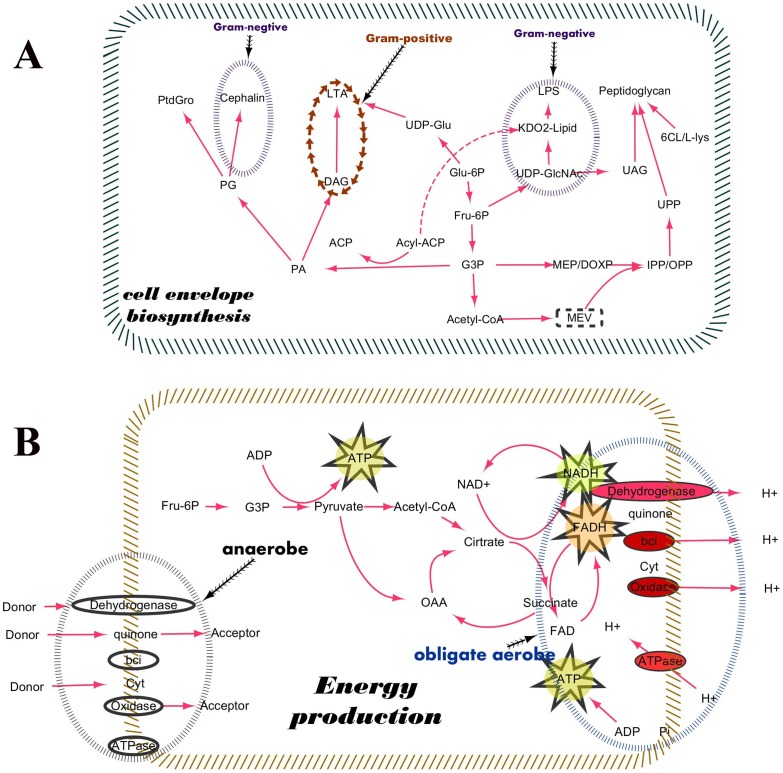
Difference in phenotype and lifestyle. **A**) Pathway differences between Gram-positive and Gram-negative bacteria. These pathways include phosphoglycerolipid and glycerolipid metabolism, terpenoid backbone biosynthesis, peptidoglycan biosynthesis, and lipopolysaccharide biosynthesis. Bacteria are categorized as either Gram-negative or Gram-positive based on differences in their cell wall compositions. The greatest difference is that Gram-negative bacteria contain an outer membrane with lipopolysaccharides, whereas lipoteichoic acid is found in the outer membrane of Gram-positive bacteria. **B**) Pathway differences between anaerobic and aerobic bacteria. Bacteria possess an important respiratory chain for energy production and maintenance of redox balance. The electron transport chains between obligate aerobic and anaerobic bacteria contain several different electron donors and acceptors. Electrons can enter the chain at three levels: a dehydrogenase, a quinone pool, or a mobile cytochrome electron carrier, all of which correspond to successively lower Gibbs free energy changes.

Essential genes are generally associated with three basic categories of essential functions or processes in organisms: maintenance of the cell envelope, energy production, and genetic information processing [37]. Apparent inconsistencies with this idea are observed between Gram-negative and Gram-positive bacteria in terms of cell wall biosynthesis (Figure 3A). Gram-negative bacteria contain an outer membrane with lipopolysaccharides (LPS), and many genes involved in LPS biosynthesis are essential genes. However, in Gram-positive bacteria, lipoteichoic acid (LTA) is present in their outer membrane, so the genes involved in LTA biosynthesis are essential. Different components of the cell wall or cell membrane correspond to various sets of essential genes; These results support the idea that the accuracy of prediction between species with similar cell wall structures is higher than that between species with different cell wall structures (i.e., Gram-positive or Gram-negative bacteria).

The transferability of essentiality annotations depends greatly on the lifestyles of organisms. We found that the essentiality of electron transport chain components varies between obligate aerobic and anaerobic bacteria because they utilize different electron donors and acceptors (Figure 3B). In our prediction, *SSA*, which is an anaerobe, exhibited good performance (AUC score  = 0.952) in predicting the essential genes of *ECO*, which is also an anaerobe, even though *ECO* is Gram-positive and *SSA* is Gram-negative. We mapped the genes to the KEGG pathway and found that the genes involved in energy production exhibited similar essentiality in *SSA* and *ECO*. For instance, the essentiality of many genes involved in electron transport chain components is consistent in the two bacteria. Similar lifestyles can decrease the discrepancy of essential gene distribution, thereby improving predictive accuracy.

The transferability of essentiality annotations between species is greatly affected by the quality of the training set, growth conditions, evolutionary distance, and lifestyle. In order to adequately improve predictive accuracy of the existing essential gene sets in new organisms, we next investigated the performance of incomplete training sets and integrated training sets.

### Incomplete training set

**Figure 4:**
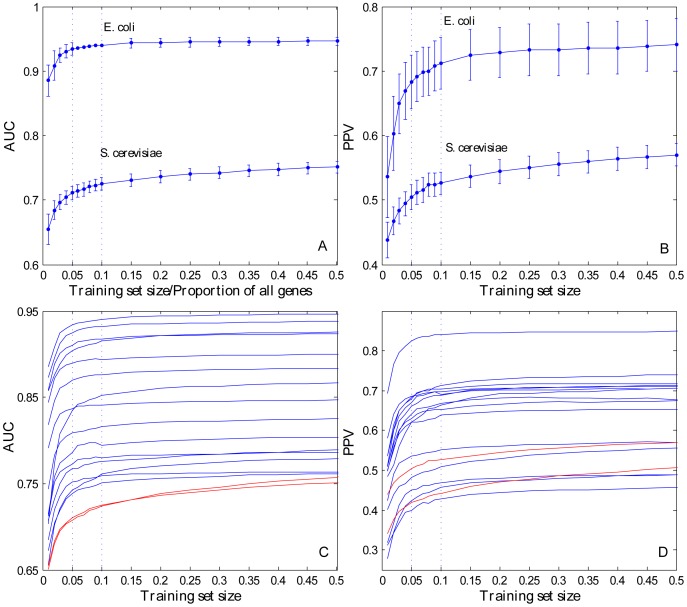
Predictive performance with different sizes of training sets. **A**) Distribution of AUC along with the different sizes of training sets in *ECO* and *SCE*. **B**) Distribution of PPV along with the different sizes of training sets in *ECO* and *SCE*. **C**) Mean curve of AUC along with the different sizes of training sets in all of the species (blue curve: 15 prokaryotes, red curve: 2 eukaryotes). **D**) Mean curve of PPV along with the different sizes of training sets in all species.

We sought to illustrate the influence of the size of a training set on essential gene prediction as follows. For species with known essential genes, 20% of the genes were randomly selected as the testing set. We gradually increased the size of the randomly selected training set from the remaining 80%. Then, we carried out the prediction and calculated AUC and PPV scores based on the training sets with different sizes. We obtained the AUC and PPV distributions and curves as well as the different sizes of training sets by simulating 1,000 replications (random selection of the training set and the testing set; Figures 4A and 4B; Table S5). The results showed that the predictive accuracy and robustness gradually improved as the size of the training set increased. In addition, the predictive accuracy improved rapidly in the previous phase (size <5%) and finally reached saturation after the size of the training set was 10% of the total genes (Figures 4C and 4D). We also found that the influence of the completeness of the training set in prokaryotes was weaker than that in eukaryotes. This result is consistent with that of a previous study in which the percentage of essential genes required to be included in the training sets in eukaryotes was reported to be twice that in prokaryotes for optimal performance [56].

### Integrated training sets

**Figure 5:**
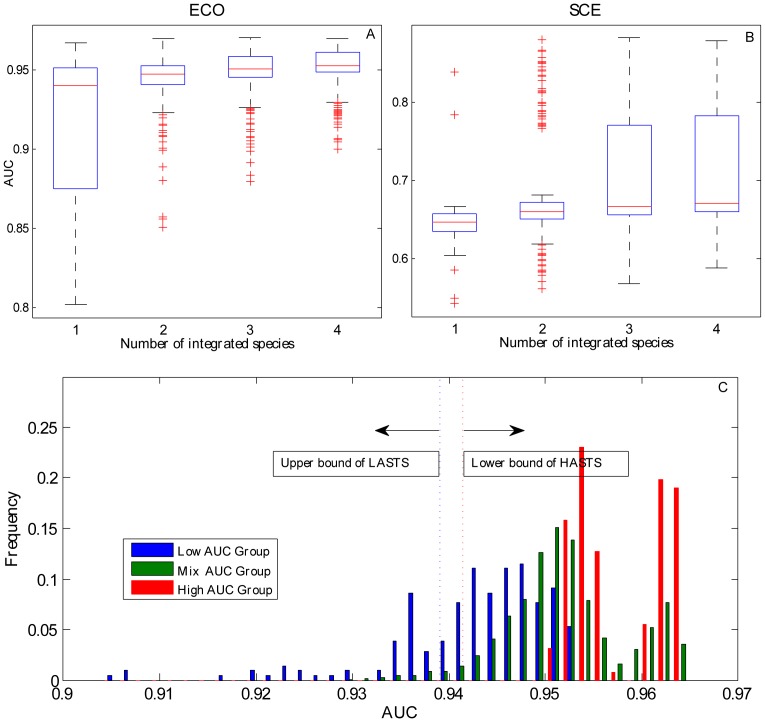
Performance of integrated training sets. **A**) Comparison of the performance of non-integrated training sets with integrated training sets in *ECO*. The boxplot with X axle 1 indicates the AUC score distribution with non-integrated training sets. The boxplots with X axles 2, 3, and 4 indicate the AUC score distributions with the integrated training sets, where 2, 3, and 4 represent integration of 2, 3, and 4 species, respectively, out of the rest of the 19 species. **B**) Comparison of the performance of non-integrated training sets with integrated training sets in *SCE*. **C**) Comparison of the difference in diverse groups of integrated training sets. The blue histogram refers to low AUC group, the green histogram refers to mixed AUC group, and the red histogram refers to high AUC group. The blue dashed line indicates the upper bound of LASTS, and all training sets in LASTS have lower AUC score than this value. The red dashed line indicates the lower bound of HASTS, and all training sets in HASTS have higher AUC score than this value.

We investigated the integrated training set containing essential and non-essential genes from more than one species. In brief, we selected one species (e.g., *ECO*) as the testing set. Then, according to full enumeration, two (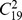 = 171), three (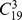 = 969), or four (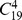 = 3876) species were chosen from the the rest of 19 species and integrated into a new training set. Subsequently, we used the new integrated training sets to construct a predictive model and compared its performance with the non-integrated training sets (the training set with only one species). Figures 5A and 5B showed the comparisons of AUC scores among the training sets (i.e., without integration, integrating two species, integrating three species, and integrating four species) when *ECO* and *SCE* were used as the testing set, respectively (for the remaining species as testing sets, see Figure S1). We observed that the prediction accuracy improved continually and significantly (t-test, p<0.01) with the number of integrated species increased in both species (i.e. *ECO* and *SCE*) used as the testing set. Similar results were obtained when other species were used as testing sets (Figures S1). We suggest that a complete and uniform essential gene set may account for the improved accuracy in the integrated training sets.

The integrated training set can also weaken the adverse effect of a training set exhibiting poor performance. For example, the AUC score of *HIN*-*SSA* is 0.781, which is remarkably lower than the accuracy of other training sets (0.851 to 0.941). Nevertheless, when we randomly integrated *HIN* with one, two, or three other species to predict the essential genes of *SSA*, the average AUC scores were 0.911, 0.924, or 0.928, respectively. This indicated that the predictive accuracy was improved by integrating *HIN* with other species. By contrast, the AUC score of *SCE*-*SPO* is 0.783, which is significantly higher than the accuracy of other training sets (t-test, p = 2.87E-12). The average AUC score was approximately 0.780 when *SCE* was integrated randomly with other species to predict the essential genes of *SPO*. The small predictive difference between the non-integrated and integrated training sets demonstrated that integrating training sets did not weaken the positive effect of training sets that exhibited excellent performance.

To further verify that integrated training sets are superior to non-integrated training set, we divided the corresponding 19 training sets (except the selected testing set) into two groups. The first group comprised nine training sets with AUC scores that exceeded a specific threshold and were defined as high AUC score training sets (HASTS). The second group comprised the remaining 10 training sets defined as low AUC score training sets (LASTS). We then selected four training sets from the HASTS and integrated them to carry out prediction. A distribution of AUC scores (high AUC group) was obtained by applying all combinations. Likewise, we established a low AUC group whose integrated training sets were obtained from the LASTS, and a mixed AUC group whose integrated training sets were obtained from both HASTS and LASTS. Figure 5C shows the distribution differences among low AUC group, mixed AUC group, and high AUC group (with *ECO* as the testing set). As expected, we observed that the high AUC group was significantly better than low and mixed AUC groups (t-test, p<1E-100). More importantly, although the low AUC group exhibited the worst performance, many predictions (158/210) in the low AUC group had higher AUC score than the specific threshold (0.939), which marks the best performance obtained using the non-integrated training sets. Conversely, few predictions in the high AUC group showed lower accuracy than the specific threshold (0.941).

We then compared the results of applying the four rules for good training sets described above with random selection of data sets for integration. First, we selected one species (e.g., *ECO*) as the testing set. Then, according to the four rules, four species (i.e. *STY*, *PAU*, *BSU*, and *CCS*, where *STY* is closely related to *ECO*) were selected for integration. All of the selected species are rod-shaped bacteria and their essential genes were obtained on nutrient-rich media. Four of them are gram-negative and facultative bacteria, and both *PAU* and *BSU* have very reliable essential gene sets. We used this integrated training set to perform predictions and obtained an AUC score which served as the threshold value. Subsequently, we randomly selected four data sets for integration and performed predictions on the same testing set (e.g., *ECO*). A p-value was then obtained as the proportion of 10,000 computer replications in which the simulated AUC scores exceed the threshold value. The distributions of the simulated AUC scores for all 20 species are displayed in Figure S2, and Table 3 shows the p-values for each species used as the testing set. All p-values were less than 0.05, demonstrating that selection of training sets based on the four rules yielded better performance than random selection.

**Table 3:** Performance of the integrated training sets under the four rules.

Testing sets	Training sets	P-values
ECO	STY, PAU, BSU, CCS	0.0080
STY	ECO, PAU, BSU, FTN	0.0037
HIN	STY, PAU, SPO, SAO	0.0024
VCH	ECO, PAU, SPO, FTN	0.0014
PAU	VCH, CCS, ECO, FTN	0.0011
ACI	PAU, SSA, ECO, CCS	0.0003
FTN	VCH, PAU, ECO, BTH	0.0048
HPY	PAU, STY, ECO, VCH	0.0013
CCS	PAU, FTN, ECO, BTH	0.0035
BSU	SAO, SPN, ECO, CCS	0.0022
SAO	SPN, BSU, SCE, ECO	0.0402
SPN	SAO, HIN, ECO, SSA	0.0095
SSA	SAO, ACI, ECO, FTN	0.0247
MGE	MPU, SAO, ECO, HIN	0.0017
MPU	MGE, SAO, ECO, FTN	0.0063
MTU	SAO, HIN, ECO, CCS	0.0034
BTH	PGN, HIN, SCE, FTN	0.0020
PGN	BTH, PAU, ECO, SAO	0.0030
SCE	SPO, MGE, ECO, HPY	0.0012
SPO	SCE, MGE, ECO, HPY	0.0147

Note: The first column indicates the species used as the testing set. The second column indicates the training sets selected according to the four criteria.

## Conclusions

We applied a machine learning approach to predict and evaluate the essential genes reciprocally among and within 21 species whose genome-wide essentiality had been determined by experimental laboratory methods. Our results showed that selection of different training sets greatly influenced predictive accuracy. We analyzed the mechanism by which training set selection affected the transferability of essentiality annotation across organisms and developed four criteria for effective training set selection.

First, essential genes in the selected training set should be reliable. High-throughput gene disruption systems (e.g., transposon mutagenesis) could improve the efficiency of essential gene identification by permitting experimental validation, but the quality of the predicted essential gene set was definitely lower than that obtained using a genome-wide set of gene deletions. Therefore, determination of essential gene sets from databases or previous studies should be done with caution before using them. Second, the growth conditions under which the essential genes were defined in the training set selection should be identified. Gene essentiality is possibly a contextual property [6], and various growth conditions correspond to different essential gene sets. Thus, essentiality should be transferred across species under the same growth conditions. Third, the species used as the training set should be closely related to the target organism. Although Deng [30] reported that gene essentiality can be reliably predicted by using features trained and tested in a distantly related organism, our results revealed significant negative correlation between predictive accuracy and divergence times across organisms. For distantly related organisms, although few good predictions were occasionally obtained, a very large variation in predictive accuracy was observed. Fourth, organisms used as training set or prediction set should have similar phenotypes or lifestyles. We found that organisms exhibiting similar lifestyles or living in the same environment (e.g., extreme environments) may share similar essential gene sets.

We next investigated the influence of incomplete training sets on predictive accuracy within species. For organisms whose essential genes are partially identified, our results showed that the best performance in predicting the rest of the genes could be achieved when at least 10% of the total essential genes was used as training set.

Aside from incomplete training sets, we also investigated the performance of an integrated training set with multiple organisms. We found that predictions based on the integrated training set were more stable and accurate than those based on a singular training set. Furthermore, compared to random selection data sets for integration, better performance was obtained when the integrated data sets were selected according to the four criteria we determined.

Thus, our study provided valuable information regarding essential gene prediction. However, our research was performed *in silico* and focused only on bacteria and fungi. Further studies should be conducted to investigate the predictability of essential genes across more complex organisms, such as plants or animals.

## Supporting Information

Table S1 **Fundamental information of the 21 species whose essential genes have been identified by different approaches.** The information included the number of essential genes, morphological characters, experimental methods and so on.
(XLS)

Table S2 **Interquartile ranges of 21 species.**
(XLS)

Table S3 **Divergence times among 21 species.**
(XLS)

Table S4 **PPV matrices corresponding to the first 100, 200, 300, and 400 genes.**
(XLS)

Table S5 **Mean and standard deviation of predictive accuracy with increasing training set size.**
(XLS)

Figure S1 **Performance of integrated training sets in the other species.** The boxplot with X axle 1 indicates the AUC score distribution with non-integrated training sets. The boxplots with X axles 2, 3, and 4 indicate the AUC score distributions with the integrated training sets, where 2, 3, and 4 represent integration of 2, 3, and 4 species, respectively.
(PDF)

Figure S2 **Distributions of the simulated AUC scores.** Each frequency histogram shows the distribution of AUC scores obtained after 10,000 simulations. The red line indicates the AUC score generated by applying the four rules.
(PDF)
