# Supplementary figures and images for: Training Set Selection for the Prediction of Essential Genes
Source: PLoS One. 2014 Jan 22;9(1):e86805. doi: 10.1371/journal.pone.0086805 (PMC3899339; doi:10.1371/journal.pone.0086805)

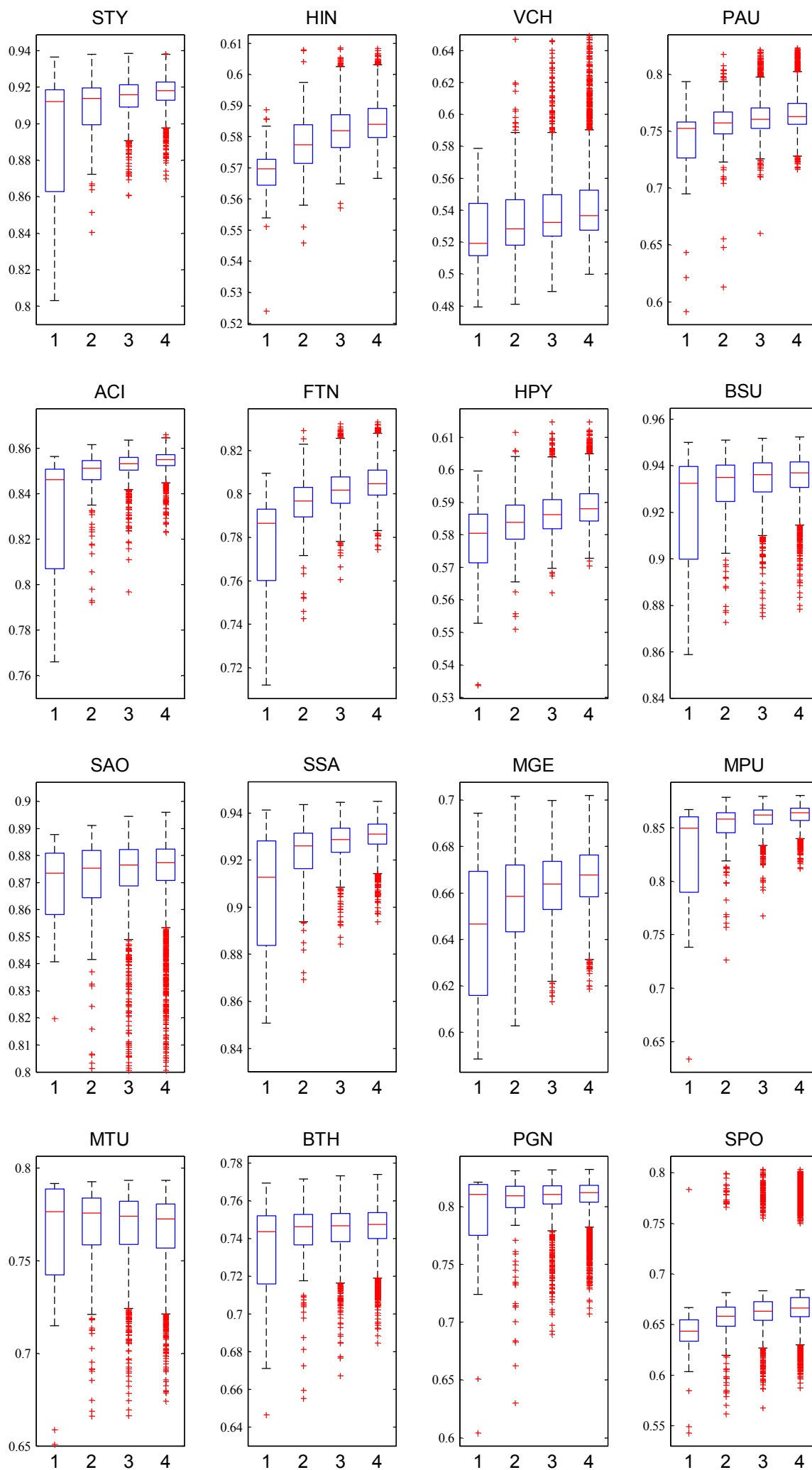

Supplement: Figure S1 — Performance of integrated training sets in the other species. The boxplot with X axle 1 indicates the AUC score distribution with non-integrated training sets. The boxplots with X axles 2, 3, and 4 indicate the AUC score distributions with the integrated training sets, where 2, 3, and 4 represent integration of 2, 3, and 4 species, respectively. (PDF) [file pone.0086805.s006.pdf]

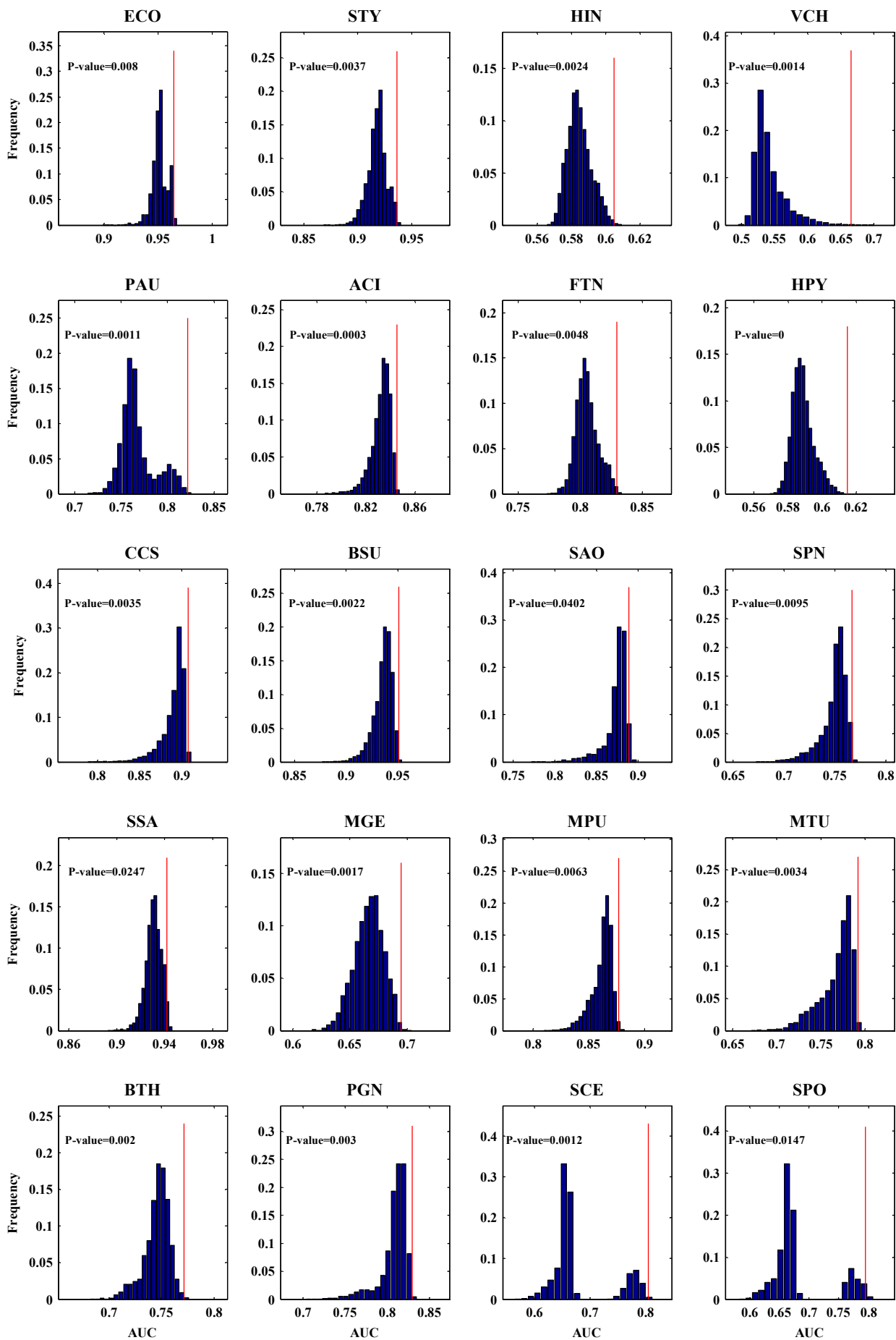

Supplement: Figure S2 — Distributions of the simulated AUC scores. Each frequency histogram shows the distribution of AUC scores obtained after 10,000 simulations. The red line indicates the AUC score generated by applying the four rules. (PDF) [file pone.0086805.s007.pdf]
